# Forecasting Patient-Specific Abdominal Aortic Aneurysm Geometry with Mixed-Effects Models

**DOI:** 10.3390/diagnostics16091409

**Published:** 2026-05-06

**Authors:** Juan C. Restrepo, Maria L. Bolanos, Seungik Baek, Satish C. Muluk, Mark K. Eskandari, Vikram S. Kashyap, Eanas Yassa, Ender A. Finol

**Affiliations:** 1 Department of Mechanical, Aerospace, and Industrial Engineering, The University of Texas at San Antonio, San Antonio, TX 78249, USA; juan.restrepo2@my.utsa.edu (J.C.R.); maria.bolanos@my.utsa.edu (M.L.B.); 2 Department of Mechanical Engineering, Michigan State University, East Lansing, MI 48824, USA; sbaek@msu.edu; 3 Department of Thoracic and Cardiovascular Surgery, Allegheny Health Network, Allegheny General Hospital, Pittsburgh, PA 15212, USA; satish.muluk@ahn.org; 4 Feinberg School of Medicine, Northwestern University, Chicago, IL 60611, USA; mark.eskandari@nm.org; 5 Cardiovascular Health, Corewell Health, Grand Rapids, MI 49503, USA; vikram.kashyap@corewellhealth.org (V.S.K.); eanas.yassa@corewellhealth.org (E.Y.)

**Keywords:** abdominal aortic aneurysms, geometry forecasting, mixed-effects, contrast-enhanced CTA, patient-specific modeling, aneurysm remodeling, heterogeneous remodeling, 3D reconstruction

## Abstract

**Background/Objectives:** Abdominal aortic aneurysm (AAA) surveillance is based largely on monitoring the maximum diameter, a single scalar metric that obscures regional remodeling and offers limited information on the location and time dependency of the growth rate. The present work addresses this limitation with a geometry-based patient-specific framework that learns local, linear evolution from longitudinal clinical imaging, yielding 3D forecasts of AAA geometry at arbitrary future times. **Methods:** Lumen and outer wall surfaces are represented on a centerline-anchored cylindrical grid, with subsequent implementation of individualized linear mixed-effects models. The model is explicitly interpretable as the fixed effects predict global trends and the random effects represent regional heterogeneity. In a multicenter cohort of 79 patients, we evaluated forecasts using spatial similarity (with the 95th percentile of the Hausdorff distance—HD95) and clinically relevant global geometric scalars such as maximum diameter and volume. **Results:** When forecasting a future AAA geometry, the model achieved sub-millimetric HD95 spatial errors and less than 6% error for the aforementioned global scalars. The model was deployed in an interactive application named the *Aneurysm Forecasting Studio*, which allows a user to visualize the AAA in an explorable forecast space. **Conclusions:** During typical clinical surveillance intervals, AAA geometric remodeling is reasonably approximated as locally linear in time, enabling transparent, fast forecasts that support surveillance optimization, threshold timing, and digital twin-based interventional planning.

## 1. Introduction

An abdominal aortic aneurysm (AAA) is a degenerative enlargement of the abdominal aorta that can progress silently to a life-threatening rupture. Often asymptomatic until rupture, the associated mortality rate approaches 90% [1], making AAA the 14th leading cause of death in the United States [2,3]. Diagnosis occurs when the aortic diameter increases by ≥50% relative to the proximal, healthy segment, typically assessed via ultrasound or computed tomography angiography (CTA). Clinical management relies on maximum diameter thresholds to guide surveillance or elective repair. The Society for Vascular Surgery (SVS) recommends 12-month imaging intervals for 4.0–4.9 cm diameters and 6-month intervals for 5.0–5.4 cm. Surgical intervention is advised at 5.0–5.4 cm for women and ≥5.5 cm for men [4].

Longitudinal studies have focused on AAA growth to improve surveillance and risk stratification. Proposed models range from linear and exponential trends to stochastic frameworks capturing growth variability [5,6,7,8,9,10,11]. However, a clear consensus on the most accurate predictive model remains elusive. Previous work quantified growth in terms of changes in maximum diameter or other morphological indicators such as tortuosity, volume, or the burden of intraluminal thrombus [12,13,14,15]. More recently, we quantified AAA growth under surveillance using 53 geometric indices and fixed and mixed-effects models, further highlighting the value of geometric descriptors beyond maximum diameter alone [16]. These indices are often correlated with rupture risk [17,18,19]. For example, a larger baseline aneurysm volume and faster volume expansion have been associated with a higher risk of rupture in follow-up imaging studies [20]. Biomechanical surrogates such as peak wall stress (PWS), wall shear stress (WSS), rupture potential index (RPI/PWRI) [21,22,23,24,25], and their use with statistical methods and machine learning networks have been proposed as more precise predictors of rupture risk and growth [26,27,28,29,30].

While predictive, existing models expose a key limitation: they distill a highly complex, three-dimensional, spatially heterogeneous remodeling process [31] into a single scalar such as maximum diameter or a limited set of global descriptors. For example, Akkoyun et al.’s exponential master curves [9] accurately predict global diameters but obscure regional heterogeneity and fail to provide the 3D shape evolution critical for patient-specific risk assessment. Do et al. modeled the aneurysm surface as a spatiotemporal random field [32]. While their Dynamical Gaussian Process Implicit Surface (DGPIS) method captures spatial evolution [32], its reliance on computationally expensive Gaussian processes and Kalman filtering limits clinical scalability.

Jiang et al. used a Deep Belief Network to predict AAA shape achieving average relative errors of 3.1% (approximately a 2 mm error) while outperforming a nonlinear mixed-effects model. However, their predictions are limited to 2D antisymmetric profile representations, which could not adequately represent aortic curvature and neck bending [33]. Similarly, Zhang et al. calibrated a constrained-mixture growth-and-remodeling model (G&R) based on centerline radii and axisymmetric assumptions [18]. This model omitted the full 3D wall geometry to avoid the considerable cost of individually calibrating tissue-level parameters. These physics-based models provide a more mechanistic description of aneurysm expansion, even allowing predictive uncertainty through Bayesian calibration [18]. However, their simplified assumptions and computational demands can limit accuracy in representing the complex evolution of the sac and restrict feasibility for clinical deployment, which underscores the need for approaches that balance realism, scalability, and interpretability.

Building on our prior geometry-based LME framework [16], we introduce a geometry-based patient-specific forecasting framework for AAA shape using simple linear mixed-effects (LME) models. We assume that, over typical surveillance intervals, AAA remodeling can be reasonably approximated as locally linear in time. From contrast-enhanced CTA scans, we derive a centerline-anchored cylindrical parameterization that places all patient follow-up geometries on a common (z,θ) grid. Subsequently, the framework fits LME models of the lumen and outer wall surfaces at each grid node to describe local linear trends in time. The predicted radii and centerline coordinates are then back-projected to forecast the AAA geometry at any target time $t^{*}$. Our goals are to: (i) demonstrate that a minimal, purely geometric input ≥2 longitudinal scans suffice to predict patient-specific AAA evolution; (ii) enable clinically useful *a priori* forecasts of unseen geometries to inform surveillance and intervention planning; and (iii) rigorously quantify fidelity and forecast accuracy in centerline and reconstructed Cartesian spaces. Evaluated on 79 AAA patients from a multi-institution cohort, this simple, robust, and interpretable framework tests the hypothesis that over typical clinical intervals AAA remodeling is well-approximated as locally linear in time, with patient-level fixed effects capturing global trends and node-level random effects capturing regional heterogeneity.

## 2. Materials and Methods

Computed tomography angiography (CTA) scans were obtained from 79 AAA patients, each with at least two imaging sessions. Data were collected retrospectively and prospectively from four institutions: Allegheny Health Network (Pittsburgh, PA, USA), Northwestern Memorial Hospital (Chicago, IL, USA), Corewell Health (Grand Rapids, MI, USA), and Seoul National University Hospital (Seoul, Republic of Korea). Institutional Review Board approval was obtained at each clinical center and all prospective participants provided informed consent prior to enrolling in the study. Data collection and processing were conducted according to all relevant ethical guidelines and regulations.

Data processing was structured into two sequential phases: (i) image segmentation and (ii) morphological representation, which encompassed (a) centerline generation and (b) three-dimensional surface reconstruction. (i) Segmentation of the lumen and outer wall boundaries was performed using a U-Net model [34], and the outlier regions were manually corrected using the AAAVasc platform. The outer wall corresponds to the outermost surface of the adventitia, whereas the lumen represents the innermost blood flow boundary (the inner intima surface in the absence of thrombus, or the inner thrombus interface when present). The segmented images were then used for two independent processes in the morphological representation (ii).

First, the centerlines (a) of the lumen and aneurysm wall were extracted from the centroids of segmented slices and smoothed using Fourier series representations. Second, three-dimensional reconstructions (b) of the lumen and outer wall were generated with the TetGen kernel implemented in AAAMesh as STL surfaces. The lumen and outer wall were modeled independently, each referenced to its respective centerline, to enable separate characterization of vessel wall remodeling and luminal reshaping associated with thrombus deposition.

### 2.1. Pre-Processing and Geometry Representation

We represented the surfaces of the outer wall and lumen using a sliced centerline-aligned parameterization in cylindrical coordinates, indexed along a resampled centerline to standardize comparisons for all patients and follow-ups. The centerlines were resampled in *K* evenly spaced stations (default K=100) using linear interpolation, creating a consistent in-patient representation for all follow-up scans. For each CTA scan, the triangulated surfaces and their centerlines were sliced by planes orthogonal to the local axial direction of the centerlines in the aneurysm sac. At each centerline station k=1,2,…,K, from the renal arteries to the iliac bifurcation, we extracted the planar intersection of the surface with the slicing plane at $z_{k}=c_{z,k}$ and assembled the corresponding Cartesian contour $S_{k}={\left(x_{i},y_{i},z_{k}\right)}_{i=1}^{N}$ after uniform resampling at N=100 points. Subsequently, each contour was translated to the local centerline origin $\left(c_{x,k},c_{y,k}\right)$ and mapped to cylindrical coordinates with θ∈[−π,π). To avoid seam artifacts at θ=±π, interpolation of radii to the grid used periodic augmentation θ↦θ±2π and natural-neighbor interpolation resulting in $C_{k}={\left(r_{i},\theta_{i},z_{k}\right)}_{i=1}^{N}$. Algorithm 1 details the slicing-based cylindrical parameterization applied to an interpolated centerline *c* and a point cloud *P*. Reverse mapping was also applied to validate and reconstruct the represented point clouds. The Cartesian slices $S_{k}$ and cylindrical slices $C_{k}$ were stored to support subsequent evaluations.
**Algorithm 1** Slicing-based cylindrical parameterization (Cartesian → cylindrical).  1:**Input:** Triangulated surface *P*; Centerline $c_{k}=\left(c_{x,k},c_{y,k},c_{z,k}\right),\;k=1,\ldots ,K$; Points per contour *N*  2:**Output:** Cartesian slices $S_{k}$; cylindrical slices $C_{k}$  3:**for** k=1 **to** *K* **do**  4:   Set $z_{k}\leftarrow c_{z,k}$.  5:   $V\leftarrow \mathrm{Find}\;\mathrm{intercept}\;\mathrm{at}\;\mathrm{level}\;z_{k}\left(P,z_{k}\right)$  6:   Resample the contour uniformly to *N* points.  7:   **Cartesian slice:** $S_{k}\leftarrow {\left(V_{i,x},V_{i,y},z_{k}\right)}_{i=1}^{N}$.  8:   Translate: ${\tilde{V}}_{i,x}\leftarrow V_{i,x}-c_{x,k}$,${\tilde{V}}_{i,y}\leftarrow V_{i,y}-c_{y,k}$ for i=1,…,N.  9:   $r_{i}\leftarrow \sqrt{{\tilde{V}}_{i,x}^{2}+{\tilde{V}}_{i,y}^{2}}$,   $\theta_{i}\leftarrow \tan^{-1}\left({\tilde{V}}_{i,y},{\tilde{V}}_{i,x}\right)$.10:   Resample the contour uniformly to *N* points with periodic augmentation.11:   **Cylindrical slice:** $C_{k}\leftarrow {\left(r_{i},\theta_{i},z_{k}\right)}_{i=1}^{N}$.12:**end for**13:**return** $S_{k},\;C_{k}$

**Figure 1:**
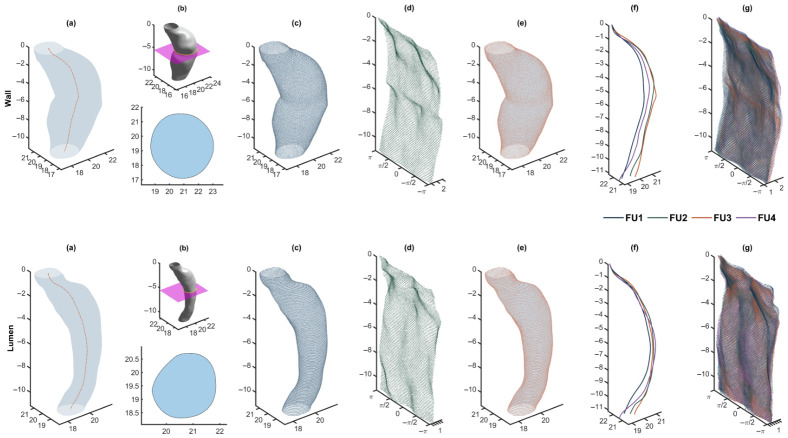
Pre-processing and geometry representation. Rows correspond to wall (top) and lumen (bottom). (**a**) Original mesh with centerline. (**b**) Exemplary slicing plane and extracted planar contours. (**c**) Intersected slicing planes overlaid on the original mesh. (**d**) Unwrapped cylindrical grid. (**e**) Cartesian reconstruction after inverse cylindrical mapping overlapping the original mesh. (**f**) Centerlines for all four CTA scans, aligned for comparison. (**g**) Unwrapped cylindrical representation for the four reconstructions; colors encode follow-up index from FU1 to FU4. Panels (**a**–**e**) show FU3.

For every CTA scan we obtain a standardized, (i) centerline-anchored parameterization shared by all imaging follow-ups. Each surface is represented on a regular K×N grid on ${\left(z_{k},\theta_{i}\right)}_{k=1}^{K}{,}_{i=1}^{N}$ (with 10,000 nodes). The axial levels $z_{k}$ and angles $\theta_{i}$ are identical for all imaging sessions for the same patient; thus, the temporal change is encoded solely in the (ii) radii on a regular grid ${\left(r_{k,i}\right)}_{k=1}^{K}{,}_{i=1}^{N}$ with a one-to-one node correspondence over time. This representation facilitates direct node-wise comparison across follow-up scans and exact inverse-mapping reconstructions for quality control, as shown in Figure 1.

### 2.2. Mixed-Effects Modeling

Using the previously calculated centerlines and radii grids, we modeled patient-specific AAA shape evolution with LME models fitted *per patient p* and *per layer* ℓ∈{L,O} (L= Lumen, O= Outer wall). Time is encoded as $t_{tp}$, the number of days since the patient’s first CTA scan (thus, $t_{0p}=0$ at baseline). Centerline and lattice nodes were indexed before model fitting to represent regional heterogeneity with nodal-specific effects (note that this indexing differs from the one used in Section 2.1). Centerline nodes were indexed by n=1,…,K and nodes in the lattice with n=1,…,KN after flattening the $\left(z_{k},\theta_{i}\right)$ grid. Let $Y_{tn\ell p}$ denote one of the following geometric response variables: a centerline coordinate (*x* or *y*) or a deployed surface radius *r*. For each patient *p* and layer *ℓ* and node *n* at time *t*, we fit the LME as follows:

(1) $$Y_{tn\ell p}=\left(\alpha_{\ell p}+b_{0n\ell p}\right)+\left(\beta_{\ell p}+b_{1n\ell p}\right)\;t_{tp}+\varepsilon_{tn\ell p}.$$

where $\alpha_{\ell p}$ and $\beta_{\ell p}$ are the *patient-level fixed* intercept and slope, and $b_{0n\ell p}$, $b_{1n\ell p}$ are the *node-level random* intercept and slope that model local deviations along the centerline or lattice within patient *p* in layer *ℓ*. We enforced uncorrelated random effects and scripted the LME model in MATLAB R2024b (The MathWorks, Inc., Natick, MA, USA): $b_{0n\ell p}∼N\left(0,\sigma_{0,\ell p}^{2}\right),\;b_{1n\ell p}∼N\left(0,\sigma_{1,\ell p}^{2}\right),\;\varepsilon_{tn\ell p}∼N\left(0,\sigma_{\varepsilon ,\ell p}^{2}\right),\;\mathrm{Cov}\left(b_{0n\ell p},\;b_{1n\ell p}\right)=0$, so that the 2 × 2 random-effects covariance is diagonal.

The models were estimated by Maximum Likelihood separately for each response and layer, so the variance components $\left(\sigma_{0,\ell p}^{2},\sigma_{1,\ell p}^{2},\sigma_{\varepsilon ,\ell p}^{2}\right)$ may differ between patients. This results in patient-specific fixed effects driving the global trend and nodal random effects accounting for heterogeneous remodeling. After fitting, a forecast at any target time $t^{★}$ requires only this time input. The LME models yield predictions for the centerline ${\left\{{\hat{x}}_{n}\left(t^{★}\right),{\hat{y}}_{n}\left(t^{★}\right)\right\}}_{n=1}^{K}$ and the unwrapped radii ${\left\{{\hat{r}}_{n}\left(t^{★}\right)\right\}}_{n=1}^{KN}$ reshaped to the fixed $\left(z_{k},\theta_{i}\right)$ grid. We further applied the inverse cylindrical mapping described in Section 2.1 to back-project ${\hat{r}}_{k,i}\left(t^{★}\right)$ about the predicted centerline, obtaining a Cartesian 3D forecast surface directly comparable with the observed geometry.

### 2.3. Evaluation

**Figure 2:**
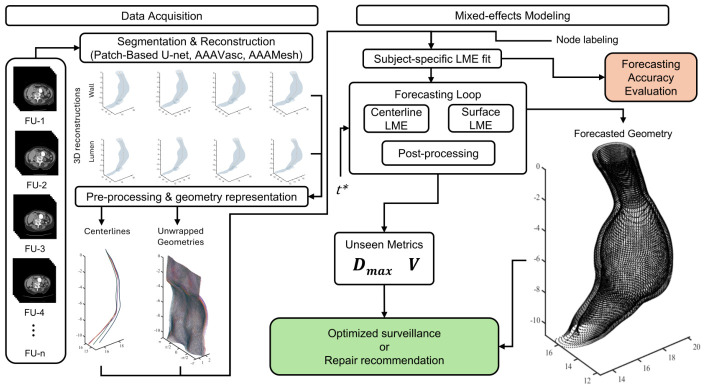
Modeling pipeline for AAA shape forecasting. The workflow integrates segmentation and 3D reconstruction, geometry representation through centerline extraction and unwrapping, and subject-specific LME fitting and evaluation. Forecasted geometries are used to derive morphological metrics to support either optimized surveillance or surgical planning.

We evaluated the accuracy of the LME models’ forecast with metrics computed separately for the outer wall and lumen, at every follow-up, using two fitting regimes: (i) fitting on all available scans and (ii) fitting after excluding the last available CTA scan for those patients with at least 3 imaging sessions in their follow-up history. This evaluation was carried out through spatial similarity using the 95th percentile of the Hausdorff Distance (HD95) between the forecast and the ground-truth anatomy, calculated in three complementary spaces: (a) the 3D centerline path (x,y,z), (b) the unwrapped (r,θ,z) point cloud, and (c) the reconstructed 3D surface (x,y,z). In each space, bidirectional nearest-neighbor distances were computed between the forecast and true point sets, and HD95 was defined as the 95th percentile of the combined distribution of the distances. This provides a robust measure of spatial agreement that discounts isolated outliers while capturing meaningful geometric discrepancies. Geometric accuracy was evaluated using geometric scalars, such as maximum diameter $\left(D_{max}\right)$ and volume (V) reported as relative percentage errors with respect to ground truth. We calculated $D_{max}$ using the hydraulic diameter to consistently quantify asymmetric 3D geometric expansions, distinguishing it from the conventional clinical interpretation based on a single orthogonal cross-sectional distance on 2D images. For each patient, we computed the aforementioned metrics at each follow-up. Our primary summaries are macro-averages of per-patient means for all follow-ups to evaluate the robustness of the patient-specific fit. Figure 2 summarizes the end-to-end workflow and highlights the evaluation endpoints.

## 3. Results

Collectively, 249 contrast-enhanced CTA data sets from 79 patients (each with ≥2 longitudinal scans) were processed following the aforementioned modeling pipeline for AAA shape forecasting. The mean number of scans per patient was 3.15 ± 1.39 (median 3; max 7). In the data set 83.5% of the patients contributed ≤4 scans, while 16.5% contributed ≥5 scans. The duration of surveillance per patient (first to last scan) averaged 2.23 ± 1.61 years, with a median of 1.69 years (IQR 1.03–2.92; range 0.38–9.11 years) and a maximum of 9.11 years (3325 days, for a single patient). For all 170 inter-scan intervals, the mean interval was 1.03 ± 0.66 years, with a median of 0.97 years (IQR 0.52–1.20; range 0.12–4.69 years). The baseline age was available for 93.7% of the patients and was 69.5 ± 7.7 years (median 70; range 51–88 years). In the cohort, 78.5% of the patients were male while 21.5% were female. No clinical co-variates were analyzed due to incomplete medical records for some of the patients.

### 3.1. Geometry Representation and Prediction with LME Models

The slicing-based cylindrical parameterization produced seam-free deployed contours and one-to-one node correspondence for all follow-ups. Back-projection checks confirmed that the inverse cylindrical mapping preserved local geometry with negligible distortion relative to the native triangulations (Figure 1e), and axial re-sampling at K=100 captured the AAA sac remodeling without aliasing. All lumen and wall centerlines were mapped to a common aligned lattice, which produced for each patient a set of resampled centerlines axially indexed by n=1,…,K and unwrapped surfaces indexed by a single node index n∈{1,…,KN} when flattened. Each observation was paired with its acquisition time *t* (days since baseline) prior to model fitting. Figure 1f illustrates the lattice-aligned centerlines while Figure 1g shows the deployed clouds for the outer wall and lumen.

**Figure 3:**
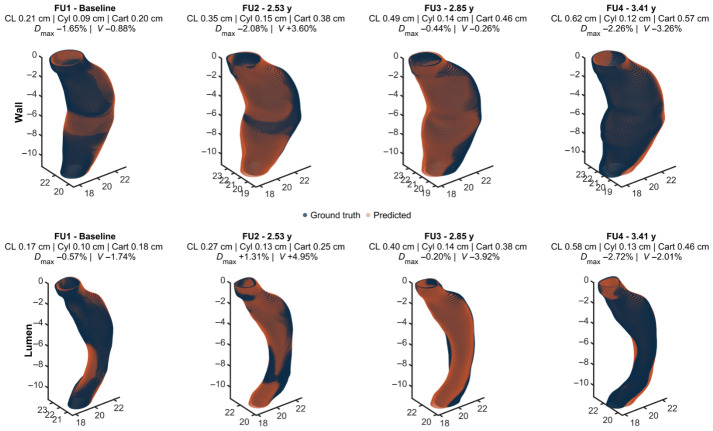
Exemplary reconstruction for a patient with four longitudinal CTA scans. Navy clouds denote ground truth geometries and orange clouds denote predictions for the outer wall (top) and lumen (bottom) surfaces. Output metrics include HD95 for the centerline (CL), cylindrical (Cyl), and Cartesian (Cart) representations, along with relative percentage differences in $D_{max}$ and *V*.

Patient-specific LME models were successfully fitted for the outer wall and lumen layers at every node and centerline station, with no convergence failures. The fitting of the model was computationally efficient, requiring between 7 and 15 s per patient using standard hardware. The mixed-effects model preserved both the global geometry and local curvature patterns in time that closely match the ground truth surfaces, as illustrated in Figure 3. In this exemplary AAA, the top row shows outer wall predictions and the bottom row lumen predictions, with columns corresponding to sequential observations over time and their associated evaluation metrics. Areas near the maximum diameter exhibited a more apparent growth, while regions near the proximal and distal ends showed less evident expansions.

### 3.2. Model Evaluation

The predictions were evaluated using macro-means, defined as the average of their mean evaluation metrics for all patients and all follow-ups. This approach quantifies how well the model fits each individual’s AAA progression, while also summarizing the overall performance of the model. The metrics included the spatial similarity measure HD95 and the percentage deviations of geometric scalars. HD95 was computed between predicted and observed geometries for three representations: (i) centerline trajectories, (ii) unwrapped point cloud, and (iii) reconstructed Cartesian representations. Geometric scalars were computed by comparing the ground-truth and reconstructed Cartesian geometries. Table 1 summarizes the quantitative evaluation metrics along with the mean predicted geometric scalars.

**Figure 4:**
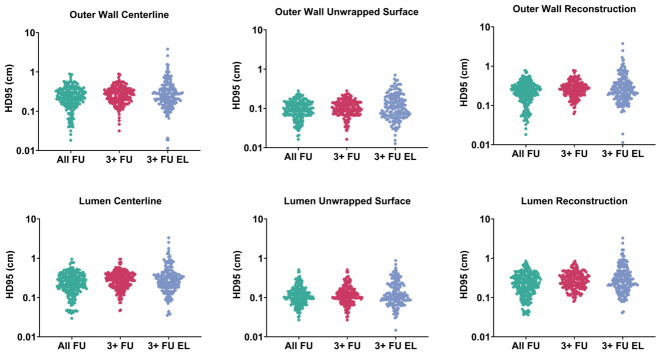
Spatial similarity for HD95 for the outer wall (**top**) and lumen (**bottom**) for all evaluation regimes. Each dot shows the per-patient mean HD95 for the centerline, unwrapped surface, and reconstructed geometry.

The model maintained a sub-millimetric mean HD95 in the cylindrical domain for the outer wall and lumen predictions within the All FU group. Diminished performance was observed for the 3+ FU groups, with the largest errors occurring when the last follow-up was excluded from training. Unwrapped surface representations consistently produced the lowest errors for both layers, confirming the deployed cylindrical coordinate system as the most stable and comparable domain for prediction. By mapping the 3D surface onto a structured grid, this parameterization reduces geometric complexity and improves spatial regularity. Furthermore, it isolates radial expansion from centerline deformation and ensures a strict one-to-one nodal correspondence across longitudinal scans, which stabilizes the linear mixed-effects estimation. The evaluation metrics for the wall centerline were slightly better than those for the lumen. Moreover, the lowest HD95 for the centerline remained higher than the maximum HD95 obtained for the unwrapped surface, indicating that the error propagated to the reconstructed Cartesian clouds, which showed values nearly identical to their corresponding centerlines. The highest HD95 values were observed for 3+ FU, as shown in Figure 4 using per-patient spatial similarity means.

**Figure 5:**
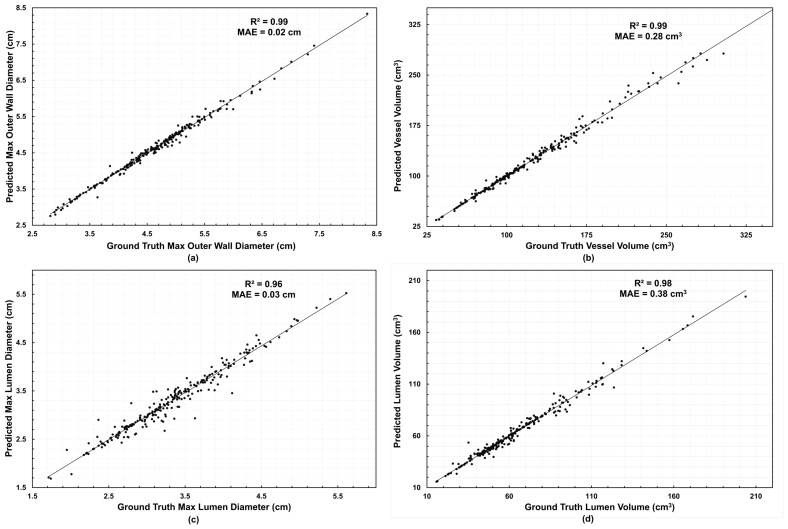
Predicted vs. ground-truth global scalars (All FU): (**a**) outer wall maximum diameter, (**b**) vessel volume, (**c**) lumen maximum diameter, (**d**) lumen volume. Data points are per patient per follow-up; $R^{2}$ and MAE are shown in each panel.

Volume and diameter errors remained below 6% for all regimes, confirming consistent forecasting performance across temporal depths and anatomical layers. The predictions of global metrics exhibited excellent correlation, with $R^{2}=0.99$ for the outer wall diameter and vessel volume, $R^{2}=0.96$ for the lumen diameter, and $R^{2}=0.98$ for the lumen volume; the corresponding values of Mean Absolute Error (MAE) were 0.02 cm, 0.28 ${\mathrm{cm}}^{3}$, 0.03 cm, and 0.38 ${\mathrm{cm}}^{3}$ respectively, confirming the model’s high predictive fidelity. The relationship between predicted and ground-truth geometric measures is shown in Figure 5.

These strong correlations demonstrate the model’s ability to reproduce global morphological trends in different anatomical layers and volumetric scales. The agreement remained high even for lumen predictions, which typically exhibit greater segmentation variability. Together, these results indicate that the model accurately generalizes aneurysm growth patterns in both the spatial and volumetric domains, maintaining a close to one-to-one correspondence between predicted and true morphometrics.

**Figure 6:**
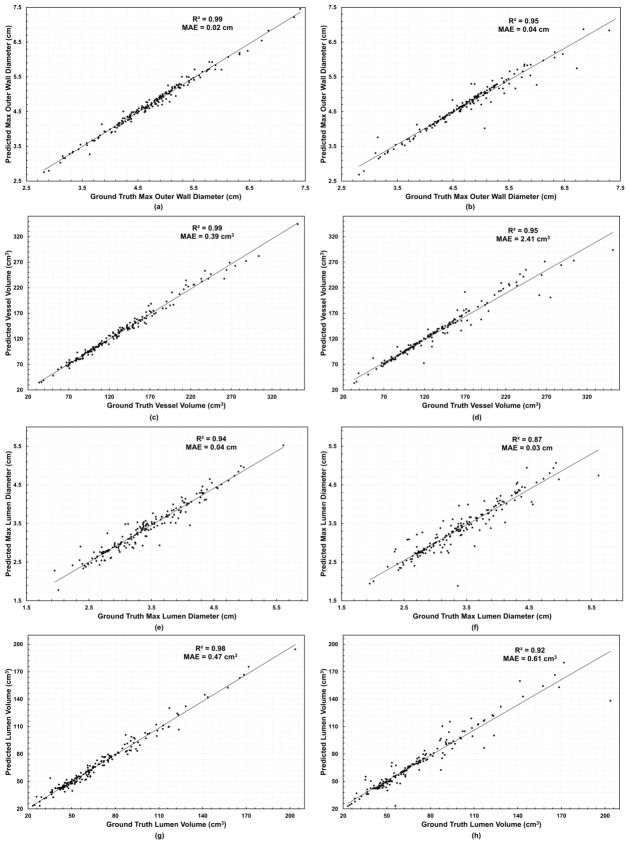
Predicted vs. ground-truth global scalars by regime. Panels: (**a**,**b**) outer wall maximum diameter, (**c**,**d**) vessel volume, (**e**,**f**) lumen maximum diameter, (**g**,**h**) lumen volume. Left column: (**a**,**c**,**e**,**g**) are the 3+ FU group metrics; right column: (**b**,**d**,**f**,**h**) are the 3+ FU EL group metrics. Points are per patient per follow-up; $R^{2}$ and MAE are shown in each panel.

We observed a decrease in performance for all evaluation metrics when the last follow-up was excluded from fitting, as shown in the 3+ FU EL subset (evaluated in a sub-sample of 43 patients with three or more longitudinal scans). Although correlations remained high, lower $R^{2}$ values were evident for both diameter and volume metrics, particularly for lumen predictions. In the 3+ FU group, $R^{2}$ values ranged from 0.94 to 0.99, with MAE varying from 0.02 to 0.04 cm for diameter metrics and from 0.39 to 0.47 ${\mathrm{cm}}^{3}$ for volume metrics. Conversely, for the 3+ FU EL group $R^{2}$ ranged from 0.87 to 0.95, and MAE ranged from 0.03 to 0.04 cm for diameter metrics and from 0.61 to 2.41 ${\mathrm{cm}}^{3}$ for volume metrics, as shown in Figure 6, reflecting the model’s sensitivity to reduced temporal information. Despite this decrease in performance, the forecasts maintained a strong one-to-one correspondence with the ground truth, confirming the model’s robustness in predicting aneurysm morphology even with limited temporal data.

### 3.3. AAA Shape Forecasting

**Figure 7:**
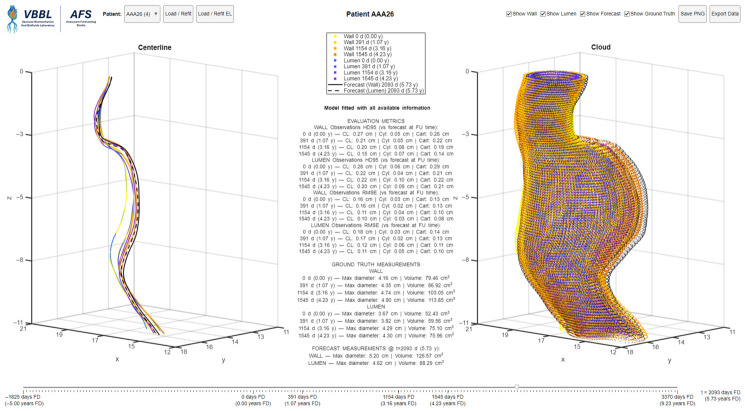
Aneurysm Forecasting Studio (AFS) showing an AAA centerline and shape corresponding to four imaging follow-ups, and a forecasted geometry 18 months beyond the last follow-up.

As an illustrative proof of concept, we deployed the models in an interactive application—the *Aneurysm Forecasting Studio (AFS)*—that turns per-patient LME fits into an explorable forecast space. As illustrated in Figure 7, AFS presents synchronized 3D centerline and cloud views, a single time slider (from 5 years pre-baseline to 5 years beyond the most recent follow-up) that updates forecasts and evaluation metrics in real time, and a panel reporting model performance. Forecasts are rendered instantly as the user moves the slider, and all visualizations and metrics are updated concurrently. These summaries provide immediate context at observed times and prospective guidance at any selected $t^{★}$.

AFS uses the same patient cohort produced by our pre-processing pipeline, ensuring perfect alignment between evaluation and exploration. A patient dropdown lists each ID with its number of follow-ups (e.g., AAA003 (4)), so data availability is visible at selection time. Two fitting regimes are available via one-click buttons: (i) *Load/Refit* trains on all available CTA imaging sessions; (ii) *Load/Refit EL* excludes the last follow-up to enable honest forecasting experiments (usable with at least 3 imaging sessions). The active regime is shown in the evaluation panel as “Model fitted with all available information” or “Model fitted excluding the last follow-up”. Ground truth follow-ups are color-coded by time: wall points sweep from yellow → orange, while lumen sweeps from blue → purple. Forecasts are drawn in black with the same encoding reproduced in the legend, such that the temporal ordering is clear. Users can independently toggle visibility via check-boxes.

AFS also includes one-click export utilities. *Save PNG* captures the full dashboard for figure-ready reporting, and *Export Data* writes a MATLAB file containing the ground truth geometries, forecasts, evaluation metrics, and the trained model for external reuse and audit. Figure 7 illustrates AFS for a patient with four longitudinal scans. The central panel reports HD95 and the root mean squared error (RMSE) per observation, and the forecasted maximum diameters and volumes at $t^{★}=5.73$ years from baseline.

### 3.4. Clinical Application by Threshold Timing and Digital Twin

AFS reports the maximum diameter and volume forecasted at the selected time $t^{★}$. By advancing $t^{★}$, AFS can identify when the AAA meets the clinical threshold for repair, enabling intervention planning and a “what-if” surveillance scenario within the same view. In the exemplary AAA illustrated in Figure 7, an 80-year-old female patient entered a surveillance program with $D_{\max}=4.16$ cm and V=79.46 ${\mathrm{cm}}^{3}$. By the fourth CTA scan (4.23 years after baseline), the size of the AAA had increased to 4.90 cm with V=113.85 ${\mathrm{cm}}^{3}$. AFS forecasted that the surgical threshold will be reached approximately five months after the last observation, with an estimated $D_{\max}=5$ cm and a growth rate of approximately 2 mm/year. The current SVS guideline intervals (annual imaging for 4.0–4.9 cm; every six months for 5.0–5.4 cm) [4] align with this trajectory; an interim follow-up could be scheduled at approximately 5 months to confirm meeting the threshold for repair and proper interventional planning. In addition, the forecasted AAA shape functions as a patient-specific digital twin for preoperative planning, multidisciplinary review, or downstream computational analyses. For example, preliminary endovascular graft planning can be performed at $t^{★}$ by extracting forecasted lumen diameters and centerline distances between the candidate landing zones to estimate device diameter and working length. This demonstrates how a geometry forecasting tool could improve AAA surveillance by not only predicting when an aneurysm will reach a critical size, but also providing a preview of the aneurysm shape at that time.

## 4. Discussion

Current surveillance and intervention thresholds for AAA clinical management are still driven exclusively by maximum diameter, a convenient but often overly simplistic metric. Despite long-standing concerns, this single scalar continues to guide decisions, even though it can underestimate the risk of rupture in small aneurysms [35] and overestimate it in large but stable ones [36]. Various approaches have emerged to refine patient-specific risk assessment by incorporating clinical factors, morphological features, wall biomechanics, and flexible growth models to accommodate irregular follow-up imaging and variability between patients. More recently, machine learning and statistical methods have been implemented with the goal of improving patient outcomes [37,38]. Nevertheless, a lack of consensus persists as to whether AAA growth should be modeled as linear or exponential. Akkoyun et al. fitted an exponential “master curve” for the maximum circumscribed spherical diameter and reported advantages over linear fits [9], while recent studies by Siika et al. and Olson et al. found that most AAAs exhibit steady, near-linear enlargement during typical monitoring periods [7,10]. In Olson’s CT surveillance study, 70% of small AAAs showed linear growth, 3% staccato growth, 4% exponential growth, and the remainder not classifiable [10]. Similarly, Siika et al. [7] observed that aneurysm diameter-based growth appeared continuous and mostly linear over approximately 5 years of surveillance in 87% of patients (mean $R^{2}=0.94$ for individual linear fits), challenging the former notion that AAA growth is highly erratic. Ristl et al. emphasized that between-patient variability and within-patient heterogeneity drive the observed distribution of growth, motivating mixed-effects formulations that yield probabilistic statements and individualized follow-up intervals [8,39]. They developed a stochastic mixed-effects model (combining geometric Brownian motion with patient-specific random effects) to capture these heterogeneities, which allowed probabilistic statements about future growth and individualized follow-up intervals with low risk of missing a rapid expansion. However, most of the aforementioned models target a single scalar endpoint, such as the maximum diameter or volume, limiting their ability to represent regional remodeling.

Efforts to model heterogeneous AAA remodeling have shown promise with physics-based approaches. A common strategy couples axisymmetric models with biomechanical growth and remodeling formulations (G&R) to embed tissue mechanics directly into forecasts. Zhang et al. used Gaussian-process priors to calibrate a constrained-mixture G&R model against centerline radii from serial CT scans, producing predictive distributions (credible bands) of future geometry that captured most withheld observations within 95% credible intervals [18]. However, their formulation relied on axisymmetric assumptions and centerline radii rather than full 3D geometries, required extensive parameter tuning including nonmeasurable tissue properties, and incurred significant computational cost per AAA, limiting its ability to model asymmetric evolution or scale clinically. To address computational demands of full 3D G&R, Jiang et al. developed a multifidelity surrogate model based on co-kriging that combines low- and high fidelity simulations, with adaptive sampling to estimate parameters efficiently. Their method provided patient-specific forecasts for 21 cases in approximately 1 to 2 h, balancing accuracy and speed [40]. Although this represents an important step forward, the reliance on complex physics-based simulations still imposes practical constraints for large-scale deployment or near point-of-care use.

Recent data-driven approaches have also advanced localized AAA forecasting. Alblas et al. developed an SE(3)-symmetric geometric deep learning framework that predicts local 3D deformation vectors directly on vascular surfaces, preserves geometric fidelity, and allows forecasts over arbitrary time intervals. Their model achieved a median diameter error of 1.18 mm and identified repair-threshold crossing within two years with an accuracy of 0.93 [41]. However, this framework depends on a substantially richer technical pipeline, including embedded multiphysical features, continuous neural-field representations, and CFD-derived hemodynamic inputs, with the hemodynamic simulations alone requiring 4 to 6 h per case on a CPU cluster in addition to GPU-based network training.

To overcome some of the aforementioned constraints, we developed a geometry-aware LME framework that assembles many small, interpretable LMEs to capture localized heterogeneous remodeling over time. By decomposing shape change into patient-level fixed effects and node-level random effects, we represent both global trends and regional deviations without committing to a unique global growth law. Model fitting was computationally efficient, requiring only 7 to 15 s per patient on standard hardware. The modular design is computationally efficient, parallelizable and explicitly links local wall and lumen changes on a patient-specific basis, serving as an AAA digital twin that forecasts future AAA morphology, providing *a priori* information to support optimized surveillance and intervention planning. The result is a fast, patient-specific modeling pipeline capable of generating geometric forecasts in seconds on standard computer hardware. We also developed a prototype interactive application named *Aneurysm Forecasting Studio (AFS)* that integrates seamlessly with automated segmentation tools, offering a practical step toward near-point-of-care AAA progression modeling. This application serves as an illustrative proof of concept rather than a systematic clinical validation. Integrating these forecasts into routine clinical workflows could directly support individualized surveillance and intervention planning.

We evaluated the performance of the LME framework with two meaningful spaces: spatial similarity, measured by HD95; and global geometric scalars, including $D_{max}$ and *V*. HD95 provides a near-worst case bound that helps assess shape completeness and ensures there are no significant local deviations, while diameter and volume align with current clinical standards of care. We used a patch-based U-Net model [34] for automatic segmentation of the lumen and outer wall boundaries, extracted centerlines, and applied a cylindrical parameterization to generate consistent cross-sections along the aneurysm over time, resulting in data well-suited for statistical learning. With a cohort assembled from four institutions, the proposed framework was exposed to realistic variability in CTA acquisition, reconstruction settings, and segmentation quality; although this heterogeneity may influence model performance, the use of a common pre-processing pipeline and the overall consistency of the forecasting results suggest that the method is reasonably robust to multicenter clinical data.

In this setup, we achieved sub-millimetric mean HD95 errors in the cylindrically parameterized domain (wall: 0.07 cm; lumen: 0.09 cm) and low scalar errors (≤2%) for $D_{max}$ and *V*, demonstrating that local linear trends are sufficient to represent patient-specific shape remodeling over typical clinical intervals. These HD95 values are in the same order of magnitude of the intrinsic spatial resolution of CTA and expected segmentation variability, typically around 0.5–1 mm, which suggests that the forecasting errors approach the practical limits imposed by the imaging and reconstruction pipeline. The high fidelity of the predictions for $D_{max}$ and *V* suggests that, for the time scales of the present study, local linear dynamics is an adequate approximation even when global diameter trajectories appear nonlinear. It should be noted that the maximum diameter and volume calculations using the lumen surface were made exclusively to assess the performance of the framework and are not equivalent to the clinically relevant $D_{max}$ and *V* calculated with the outer wall surface. Nevertheless, knowledge of lumen diameter and volume may be beneficial for pre-interventional endovascular graft repair planning.

Our approach is complementary among existing modeling strategies. Similar to other patient-specific growth modeling efforts, it is designed with clinical timelines and interpretability in mind. Compared to Bayesian G&R [18], it operates directly with native AAA geometry to predict spatial remodeling over time and, as multifidelity surrogates, it emphasizes scalability and computational efficiency [40,41]. Our contribution builds on these ideas by offering a computationally lean, geometry-first alternative that requires only the segmented AAA shape and timestamps (with no material parameter estimation or volume meshing) and runs in seconds on standard computer hardware. This structure enables high-fidelity, patient-specific surface forecasts from which clinical scalars emerge as by-products, while remaining flexible enough to integrate with more intensive biomechanical analyses if needed. In doing so, our workflow provides a pragmatic path for justifying patient-specific morphology forecasting that reflects the spatial complexity of AAA shape remodeling, which could complement biomechanical analyses and provide a clinically feasible tool for predicting future geometric features.

With the current trend toward individualized AAA clinical standards of care, we expect that fast and interpretable models such as those described in the present work, in conjunction with advances in biomechanics and machine learning, will play an increasing role in risk stratification and treatment planning for AAA patients. For example, biomechanical analyses such as wall stress computation or fluid–solid interaction (FSI) modeling could be applied to the forecasted geometries to estimate rupture risk surrogates (e.g., PWS, PWRI) at future times. This integration would enable a truly predictive and patient-specific risk assessment pipeline. Such a framework is expected to help vascular surgeons use statements such as “if we wait one year, the aneurysm is expected to reach X cm and Y stress, implying a Z % annual rupture risk” to effectively treat AAA patients. Incorporating such analyses in a timely manner represents an important step toward achieving a comprehensive AAA digital twin.

The proposed LME shape forecasting framework is subject to several important limitations:

1. **Longitudinal deformation along the axial z axis is not explicitly modeled:** The evolution of AAA geometry is assumed primarily to occur in a cross-sectional caliber and shape.
2. **Spatial coupling across nodes is not enforced:** Node-level random effects are treated as independent for spatial positions and layers (lumen and outer wall). This choice simplifies computation and enables parallel fitting, but may produce small, locally inconsistent fluctuations between neighboring nodes and does not enforce coherence between lumen and wall when local noise or registration errors are present. Future work should address these limitations by introducing spatial structure (e.g., Gaussian-process or spline priors over (z,θ), random-walk smoothness penalties, or block-covariance terms coupling lumen and wall), thereby improving anatomical plausibility while preserving patient-level trends.
3. **Overfitting in cases with only two longitudinal imaging sessions per patient:** In such instances, overfitting is expected because the model interpolates linearly between the two time points. Consequently, the forward and backward forecasts are based on linear extrapolations of the observed behavior, which may underestimate or overestimate growth if the unknown AAA shape evolution is curvilinear, limiting their robustness and increasing uncertainty. From a statistical modeling perspective, there is no single optimal number of time points; rather, the predictive reliability of the linear mixed-effects model continuously improves as more longitudinal observations become available to inform the patient-specific morphological growth behavior. A minimum of three time points is required to represent the true variance in the growth trajectory beyond strict linear interpolation. However, our holdout results (Figure 6) suggest that with just two time points, the framework can give a reasonable short-term forecast, but evidently more data per patient yields greater stability and confidence in the shape forecasting.
4. **Irregularly spaced observations:** Due to the nature of the data used in the study, irregularly spaced observations introduce bias into the estimated linear trend over specific temporal windows. Equally spaced follow-up intervals are considered optimal, as they prevent the model fitting from being disproportionately weighted by clustered imaging sessions.
5. **Absence of clinical covariates:** Due to incomplete medical records, clinical covariates (e.g., sex, smoking status, hypertension, and BMI) were not included in the current modeling framework. Future work incorporating these variables into the fixed-effects structure could improve predictive accuracy, refine growth trends, and enhance patient-specific surveillance.

## 5. Conclusions

The present work described a simple, effective, and fast approach for patient-specific AAA geometry forecasting using linear mixed-effects models on centerline-aligned surface grids. Despite its simplicity, the model achieved excellent accuracy, sub-millimetric spatial errors, and low percentage errors in key maximum diameters and volumes while predicting future aneurysm shapes beyond typical surveillance intervals. The proposed framework is highly parallelizable and interpretable, providing an instantly updatable digital twin of a patient’s AAA that can assist in surveillance optimization, threshold timing, and preoperative planning. Given its minimal requirements (past imaging scans and desired forecast time) and real-time performance, the framework can be readily integrated into clinical practice to enhance AAA management and provide individualized pathways for optimized surveillance or repair recommendations.

## Figures and Tables

**Table 1:** Quantitative evaluation of model performance for all regimes and representations. All FU: evaluation for 79 patients using all available observations. 3+ FU: evaluation for 43 patients with three or more follow-ups. 3+ FU EL: same cohort excluding the last follow-up from fitting.

Layer	Regime	Representation					Mean Predicted Geometric Scalars	
		Spatial Similarity HD95 (cm)			Geometric Scalar Error (%)			
		Centerline	Unwrapped Surface	Reconstruction	Max Diameter	Volume	Max Diameter (cm)	Volume (${\mathrm{cm}}^{3}$)
**Wall**	**All FU**	0.19	0.07	0.19	0.90%	1.64%	4.45	122.14
	**3+ FU**	0.29	0.11	0.28	1.42%	2.99%	4.69	128.15
	**3+ FU EL**	0.36	0.12	0.37	1.82%	3.83%	4.67	126.88
**Lumen**	**All FU**	0.21	0.09	0.21	2.27%	2.68%	3.27	62.38
	**3+ FU**	0.30	0.13	0.30	3.28%	4.66%	3.34	66.14
	**3+ FU EL**	0.38	0.14	0.38	3.97%	5.96%	3.35	66.01

## Data Availability

The data set used in this study is not publicly accessible due to regulations imposed by Institutional Review Boards of the respective clinical centers. However, it can be obtained by making a reasonable request to co-author E.A.F. The AFS is available from J.C.R. and E.A.F. upon request.
